# The effect of feeding a low iron diet prior to and during gestation on fetal and maternal iron homeostasis in two strains of rat

**DOI:** 10.1186/1477-7827-11-32

**Published:** 2013-05-01

**Authors:** Ruth Cornock, Lorraine Gambling, Simon C Langley-Evans, Harry J McArdle, Sarah McMullen

**Affiliations:** 1 School of Biosciences, University of Nottingham, Sutton Bonington Campus, Loughborough, LE12 5RD, UK; 2 Rowett Institute of Nutrition and Health, University of Aberdeen, Aberdeen, AB21 9SB, UK

**Keywords:** Pregnancy, Iron, Placenta, Liver, Maternal, Fetus, Development, Rat

## Abstract

**Background:**

Iron deficiency anaemia during pregnancy is a global problem, with short and long term consequences for maternal and child health. Animal models have demonstrated that the developing fetus is vulnerable to maternal iron restriction, impacting on postnatal metabolic and blood pressure regulation. Whilst long-term outcomes are similar across different models, the commonality in mechanistic events across models is unknown. This study examined the impact of iron deficiency on maternal and fetal iron homeostasis in two strains of rat.

**Methods:**

Wistar (n=20) and Rowett Hooded Lister (RHL, n=19) rats were fed a control or low iron diet for 4 weeks prior to and during pregnancy. Tissues were collected at day 21 of gestation for analysis of iron content and mRNA/protein expression of regulatory proteins and transporters.

**Results:**

A reduction in maternal liver iron content in response to the low iron diet was associated with upregulation of transferrin receptor expression and a reduction in hepcidin expression in the liver of both strains, which would be expected to promote increased iron absorption across the gut and increased turnover of iron in the liver. Placental expression of transferrin and DMT1+IRE were also upregulated, indicating adaptive responses to ensure availability of iron to the fetus. There were considerable differences in hepatic maternal and fetal iron content between strains. The higher quantity of iron present in livers from Wistar rats was not explained by differences in expression of intestinal iron transporters, and may instead reflect greater materno-fetal transfer in RHL rats as indicated by increased expression of placental iron transporters in this strain.

**Conclusions:**

Our findings demonstrate substantial differences in iron homeostasis between two strains of rat during pregnancy, with variable impact of iron deficiency on the fetus. Whilst common developmental processes and pathways have been observed across different models of nutrient restriction during pregnancy, this study demonstrates differences in maternal adaptation which may impact on the trajectory of the programmed response.

## Background

The World Health Organisation estimates that anaemia affects nearly two billion people worldwide, with iron deficiency anaemia accounting for 75-80% of the total burden [1,2]. Iron deficiency is most common in infants, adolescent girls and women of child bearing age [3] and the prevalence during pregnancy is a global problem, ranging from 25% in Europe and the Americas to 48% and 57% in East Asia and Africa respectively [2]. It is of particular importance during pregnancy, as anaemia can result in preterm delivery of the fetus, low birth weight [4] and increased perinatal mortality [5]. In addition, iron deficiency during pregnancy has been shown to alter placental vascularisation [6] and cytokine expression [7] in the rat, and low maternal haemoglobin has been linked with the formation of an enlarged placenta in humans [8].

Iron deficiency during pregnancy has also been suggested to have longer-term consequences. In human populations, low maternal haemoglobin during pregnancy has been associated with high blood pressure in the resulting offspring during childhood [9]. In animals, long-term effects of exposure to a prenatal low iron diet include raised blood pressure [10-13], altered renal morphology and nephron number in the offspring [14,15] and altered lipid and glucose homeostasis [13]. Studies regarding the effect of iron deficiency during pregnancy on postnatal blood pressure have been carried out in rats of the Wistar strain [12] and Rowett Hooded Lister strain (RHL) [11], with the offspring of both strains exhibiting raised blood pressure in adulthood when exposed to a prenatal low iron diet.

Rodent models of nutritional insult during pregnancy are extensively used to investigate phenomena associated with programming of physiology and disease [16]. It is widely assumed that data obtained from rat and mouse studies are broadly comparable and little consideration has been given to variation between strains within a species. Earlier work reporting long-term effects of iron deficiency during pregnancy have used different strains (RHL [11,17], Wistar [12], Sprague Dawley [10]). Whilst the long-term outcomes for the developing fetus are similar in terms of raised blood pressure, it is not known whether these programmed attributes arise in response to similar maternal adaptations to the nutritional insult across all strains. This knowledge is required to improve understanding of the mechanisms of nutritional programming. Previous work in the RHL strain identified alterations in iron homeostasis in response to a low iron diet during pregnancy [17,18]. This present study aimed to extend these findings to the more widely used Wistar strain of rat, and to examine any differences between the strains in terms of iron homeostasis and response to a low iron diet.

Attention was focussed on iron uptake (involving the transferrin receptor, TfR), transport within the cell (involving the divalent metal transporter DMT1+IRE), iron export (involving ferroportin) and a protein involved in regulation of these transporters (hepcidin). Finally, expression of ferroxidase molecules (ceruloplasmin and hephaestin) was examined due to their importance in the oxidation of Fe^2+^ to Fe^3+^ to enable iron to be carried in the circulation attached to transferrin. It was expected that the feeding of a low iron diet prior to and during pregnancy would impact on the iron status of pregnant rats and their fetuses, producing an anaemic state. It was hypothesised that the placental expression of proteins associated with iron transport from the pregnant rat to fetus would be altered in both strains of rat. It was further hypothesised that iron deficiency would influence the transfer of iron between mother and fetus across the placenta.

## Methods

### Animal procedures

All animal experiments were performed under license from the United Kingdom Home Office in accordance with the 1986 Animals (Scientific Procedures) Act and following approval from the University of Nottingham Ethics Committee. Virgin female Wistar (n=20) and RHL (n=20) rats were fed either a control iron (50 mg/kg) or a low iron (7.5 mg/kg) diet from weaning and for 4 weeks prior to mating. Animal procedures in the Wistar strain were performed in the BioResources Unit at the University of Nottingham, whilst experiments in the Rowett Hooded Lister strain were performed at the Rowett Institute of Nutrition and Health (RINH), Aberdeen. All experimental diets were prepared at RINH as previously described [11] and fed *ad libitum*. The animals were group housed in cages under constant temperature and humidity, and under the same conditions at both sites. Controlled illumination with a 12 h light–dark cycle was maintained to assure regular oestrous cycles. Mating was confirmed by the presence of a semen plug found on the cage floor (gestational day 0), and dietary intervention continued throughout gestation. Dams were weighed daily and their feed intake recorded during the whole experimental period. Dams were euthanased on day 21 of gestation by cervical dislocation under terminal anaesthesia with isoflourane, and fetuses by cervical dislocation. Fetuses were counted, sexed and individually weighed. Placentas and fetal organs were also individually weighed. Placentas and maternal and fetal livers were removed, weighed and snap frozen in liquid nitrogen, and stored at −80°C until analysis. Fetal sex was determined, and three males and three females (where possible) were pooled to provide a pooled male and a pooled female sample for each litter. This ensured that there was sufficient tissue within each sample to be used for all subsequent analyses.

Duodenal samples were collected from non-pregnant female Wistar and RHL rats maintained on the control diet for a period of 6 weeks from weaning. All other conditions were the same as described previously. Following euthanasia by cervical dislocation under terminal anaesthesia with isoflourane, the gastrointestinal tract was excised and a 10 cm section of duodenum removed from immediately downstream of the pyloric sphincter. This section was sliced longitudinally into two, snap frozen in liquid nitrogen, and stored at −80°C until analysis.

### Tissue iron determination

Determination of tissue iron content was carried out at the Rowett Institute of Nutrition and Health as previously described [7]. Briefly, iron content was determined by graphite-furnace atomic spectrophotometry. To distinguish between haem and non-haem iron, samples were treated with 20% tri-chloroacetic acid.

### RNA extraction and real-time RT-PCR

All mRNA expression analyses were conducted at the University of Nottingham. RNA was extracted from snap-frozen placenta and liver tissue by the TRIzol procedure (Invitrogen, UK) and subjected to DNAse treatment (Promega, UK), phenol-chloroform extraction and ethanol precipitation. RNA was reverse transcribed using MMLV reverse transcriptase (Promega, UK). Real-time PCR primers for TfR, DMT1+IRE and hepcidin have previously been described [17,18]. Primer sequences for ceruloplasmin, hephaestin and β-actin were designed using Primer Express (v1.5; Applied Biosystems) and were as follows: ceruloplasmin [GenBank NM_012532] forward primer 5’-GAGACAAAGTTTCTGTTACGTAAAGA-3’, reverse primer 5’-GGTAGATGGCCCCCTCG-3’; hephaestin [GenBank NM_133304] forward primer 5’-TCCAATCGAATGCATGCT-3’, reverse primer 5’-AACATAACCCCATGTACA-3’; β-actin [genBank NM_031144] forward primer 5’-TTCAACACCCCAGCCATGT-3’, reverse primer 5’-GTGGTACGACCAGAGGCATACA-3’; IREG [genBank NM_133315] forward primer 5’-CATAATCTCCGTCAGCCTGCT-3’, reverse primer 5’-CACAGTCAAATCAAAGGACCAAAG-3’. Primers were purchased from MWG Biotech, Germany. Real time PCR was performed on the Lightcycler 480 system (Roche, UK). Placental mRNA expression data was normalised to β-actin expression. The expression of a number of housekeeping genes (β -actin, GAPDH and cyclophilin A) were found to be influenced by experimental conditions in maternal and fetal liver. Therefore, expression data in these tissues was normalised to the quantity of cDNA present in each sample, indicated by the fluorescence at 80°C [19]. Briefly, Oligreen stock solution (Invitrogen, UK) was diluted 1:200 in 1xTE buffer to produce a working solution, 5 μl of which was mixed with an equal volume of sample in a 384 well plate, in duplicate and the fluorescence measured at 80°C using the Lightcycler 480 system (Roche).

### Western blotting

All protein expression analyses were conducted at the University of Nottingham. Tissues were homogenised in an extraction buffer containing 50 mM Tris/HCL and 5 mM EDTA. Protein concentration was determined by the Bradford assay [20]. Samples were diluted with an equal volume of loading buffer [4% (w/v) SDS, 125 mM Tris/HCl pH 6.8, 20% (v/v, 87%) glycerol, 0.1M dithiothreitol], and adjusted to equal protein concentration. Samples were then heated at 90°C for 5 minutes before being run on SDS-polyacrylamide gels. Electrophoresis was carried out in a 10× Tris/glycine/SDS running buffer (National Diagnostics, USA). Following electrophoretic separation, proteins were transferred to nitrocellulose membrane (GE Healthcare, UK). Blots were probed with the following anti-rat antibodies: TfR diluted 1:1000 (Serotec) for both fetal liver and placental samples, ferroportin (Alpha diagnostics) diluted 1:30,000 for placental samples and 1:700 for fetal liver samples, Tubulin (Abcam, UK) diluted 1:60,000 for both fetal liver and placental samples. Blots were developed using ECL reagents and treated with goat anti-rabbit (ferroportin and tubulin) or sheep anti-mouse (TfR) horse radish peroxidise linked secondary antibody (GE Healthcare). Blots were exposed to Hyperfilm ECL (GE Healthcare) and protein bands quantified using a Quantity-One Multi Analyst system (BioRad, UK). Protein expression was normalised to tubulin expression.

### Statistical analysis

Data were analysed using SPSS (v15) and by carrying out an ANOVA or independent samples T-test where appropriate. All fetal data were analysed by three-way ANOVA to assess the effects of diet, strain and sex. The only exception to this is for the fetal liver protein expression data, where a significant difference in expression of the housekeeping protein was found between strains. Wistar and RHL data was therefore analysed separately for these two variables. There was no effect of fetal sex on any of the parameters examined in either strain or interaction of fetal sex with any of the other factors being examined. Data is therefore presented for male and females combined. Some litters contained less than three males or females and so did not provide sufficient fetal material for all analyses. The number of samples in each analysis is therefore variable. No data was actively excluded for any reason other than a missing value.

## Results

### Maternal and fetal weights

RHL rats had a significantly lower body weight at mating (P<0.05) and a significantly higher pregnancy weight gain (P<0.05) compared to Wistar rats (Table 1), There was no effect of the low iron diet on weight at mating or weight gain during pregnancy in either strain. Litter size was unaffected by strain or diet. There was a significant interaction between the effects of diet and strain on average birth weight (P<0.05, Table 1), with a low iron diet significantly reducing birth weight in the RHL strain only. Placental weight was significantly lower in the Wistar strain (P<0.05, Table 1), but was unaffected by diet in either strain.

**Table 1:** Pregnancy outcomes

	**RHL**		**Wistar**	
	**Control**	**Low iron**	**Control**	**Low iron**
	**n=11**	**n=8**	**n=10**	**n=10**
Maternal body weight at mating (g)	204.4 ± 3.7^b^	203.0 ± 3.5^b^	224.2 ± 4.3^a^	225.7 ± 6.1^a^
Pregnancy weight gain (g)	146.0 ± 5.2^a^	136.7 ± 5.8^a^	107.9 ± 4.7^b^	106.2 ± 4.9^b^
Litter size	13.2 ± 0.8	13.6 ± 1.2	11.2 ± 0.7	12.0 ± 0.9
Mean fetal weight (g)	4.7 ± 0.1^a^	4.0 ± 0.2^b^	4.5 ± 0.1^a^	4.6 ± 0.1^a^
Mean placental weight (g)	0.51 ± 0.01^a^	0.55 ± 0.04^a^	0.48 ± 0.04^b^	0.43 ± 0.02^b^

Pregnancy weight gain, litter size and fetal and placental weights in Rowett Hooded Lister (RHL) and Wistar rats exposed to a control or low iron diet prior to and during pregnancy. Data are presented as means ± s.e.m. (a>b, P<0.05).

### Maternal and fetal liver and placental iron content

**Figure 1:**
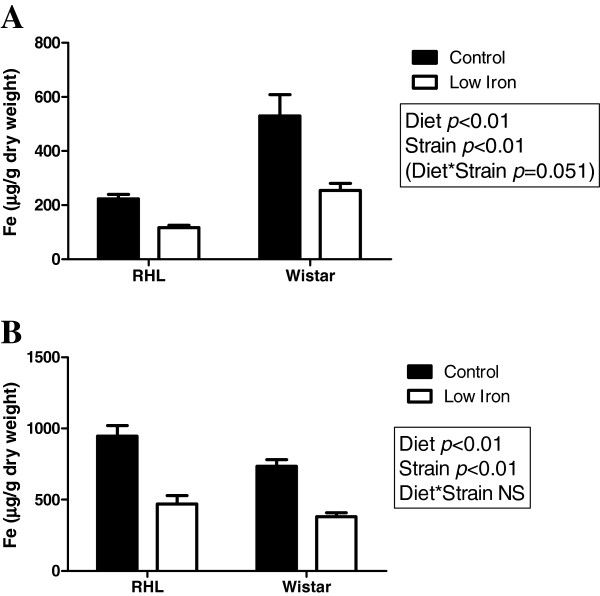
**Maternal and fetal liver iron content.** Maternal (**A**) and fetal (**B**) liver iron content at day 21 of gestation in Rowett Hooded Lister (RHL) and Wistar rats exposed to a control or low iron diet prior to and during pregnancy. Data are presented as means ± s.e.m. for n = 8–11 per group.

In both strains of rat, the iron content of the maternal liver was significantly lower in animals fed a low iron diet (diet effect: P<0.001, Figure 1A), being decreased by 52% in Wistar rats and 48% in RHL rats. Despite being fed an identical diet, Wistar rats had a greater maternal liver iron content than RHL rats (strain effect: P<0.001, Figure 1A), to the extent that iron-depleted Wistar rats had a higher liver iron content than RHL controls. Similarly, the iron content of the fetal liver was significantly lower in animals exposed to a low iron diet *in utero* (diet effect: P<0.01, Figure 1B), being decreased by 51% in both strains of rat. In contrast to the maternal livers, Wistar fetuses had significantly *lower* liver iron content than those of the RHL strain (strain effect: P<0.01, Figure 1B).

**Figure 2:**
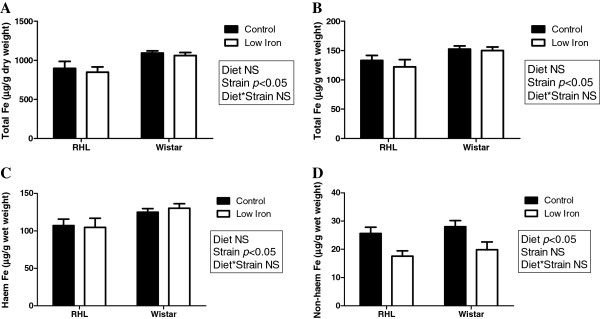
**Placental iron content.** Placental total iron (**A** and **B**), haem iron (**C**) and non-haem iron (**D**) content in Rowett Hooded Lister (RHL) and Wistar rats exposed to a control or low iron diet prior to and during pregnancy. Data are presented as means ± s.e.m. for n = 14–21 per group.

Placentas of Wistar rats had significantly greater total iron content than RHL rats (29%, P<0.05, Figure 2A & B). This was reflected in a greater haem iron (strain effect: P<0.05, Figure 2C), but not non-haem iron (Figure 2D) content. The non-haem iron content of the placenta was significantly reduced in animals fed a low iron diet (diet effect: P<0.05, Figure 2D), irrespective of strain.

### Maternal liver iron homeostasis

**Figure 3:**
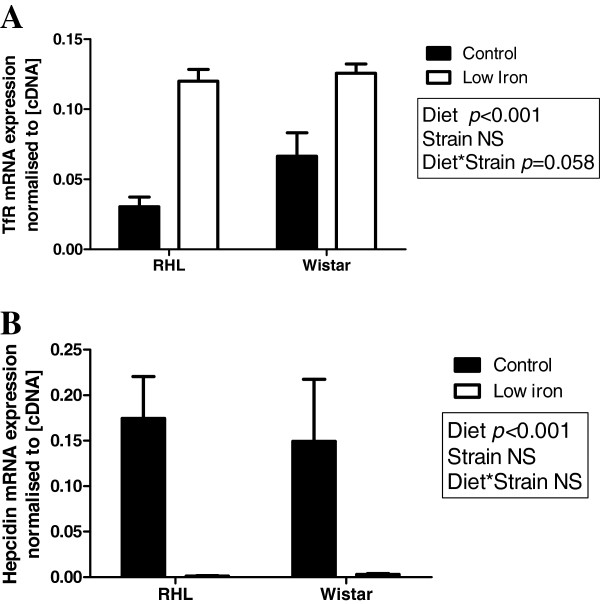
**Maternal liver TfR and hepcidin expression.** Maternal liver TfR (**A**) and hepcidin (**B**) mRNA expression in Rowett Hooded Lister (RHL) and Wistar rats exposed to a control or low iron diet prior to and during pregnancy. Data are presented as means ± s.e.m. for n = 8–11 per group. Expression is normalised to cDNA fluorescence at 80°C.

Expression of TfR mRNA in the maternal liver was significantly greater in animals fed a low iron diet prior to and during gestation (Figure 3A, diet effect: P<0.001), with expression tending to be higher in Wistar compared to RHL rats (Figure 3A, strain effect: P=0.058). Expression of hepcidin mRNA in the maternal liver was significantly reduced in rats fed a low iron diet prior to and during gestation to the extent that expression was barely detectable (Figure 3B, diet effect: p<0.001). There was no effect of strain on hepcidin mRNA expression (Figure 3B). There was no effect of diet or strain on the mRNA expression of ceruloplasmin in the maternal liver (data not shown).

### Fetal liver iron homeostasis

**Figure 4:**
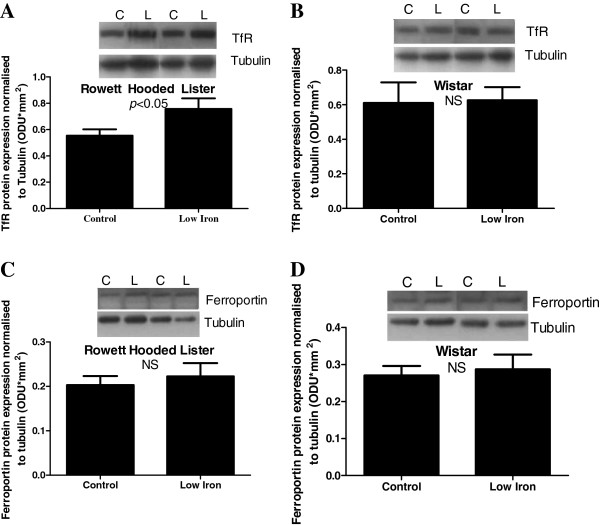
**Fetal liver TfR and ferroportin expression.** Fetal liver protein expression of TfR (**A** &**B**) and ferroportin (**C** &**D**) proteins in Rowett Hooded Lister (**A** &**C**) and Wistar (**B** &**D**) rats exposed to a control or low iron diet prior to and during pregnancy. Data are presented as means ± s.e.m. for n = 16–19 per group. Protein expression is normalised to tubulin expression. Representative sample blots are shown.

**Figure 5:**
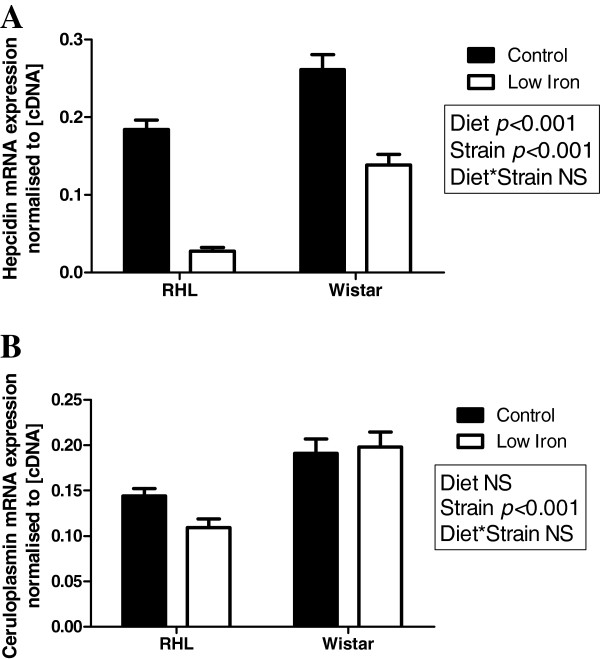
**Fetal liver hepcidin and ceruloplasmin expression.** Fetal liver mRNA expression of hepcidin (**A**) and ceruloplasmin (**B**) in Rowett Hooded Lister (RHL) and Wistar rats exposed to a control or low iron diet prior to and during pregnancy. Data are presented as means ± s.e.m. for n = 16–19 per group. Expression is normalised to cDNA fluorescence at 80°C.

Expression of TfR protein in the fetal liver was significantly increased by exposure to a low iron diet in RHL rats (Figure 4B, P<0.05), but not Wistar rats (Figure 4A). Ferroportin protein expression was not affected by diet in either strain (Figure 4C & D). A comparison between strains could not be made for the expression of TfR or ferroportin, as tubulin expression differed between strains. Fetal liver hepcidin mRNA expression was significantly higher in Wistar rats compared to RHL rats (Figure 5A, 43%, P<0.001), and was significantly decreased by exposure to a maternal low iron diet in both strains (Figure 5A, 61%, P<0.001). Fetal liver ceruloplasmin mRNA expression was significantly higher in Wistar rats compared to RHL rats (Figure 5B, 52%, P<0.001), but was not affected by diet in either strain.

### Placental iron transport

**Figure 6:**
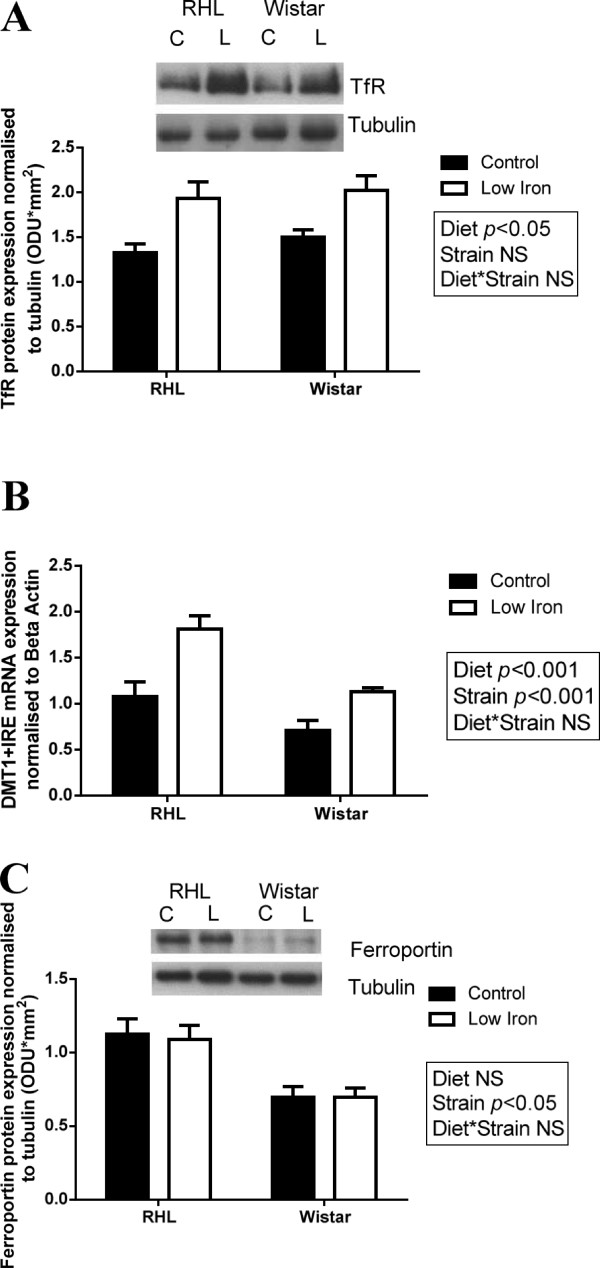
**Placental TfR, DMT1+IRE and ferroportin expression.** Placental expression of TfR protein (**A**), DMT1+IRE mRNA (**B**) and ferroportin protein (**C**) in Rowett Hooded Lister (RHL) and Wistar rats exposed to a control or low iron diet prior to and during pregnancy. Data are presented as means ± s.e.m. for n = 15–21 per group. Protein expression is normalised to tubulin expression and mRNA expression is normalised to β-actin expression. Representative sample blots are shown.

Placental TfR protein expression was significantly higher in rats fed a low iron diet prior to and during gestation (Figure 6A, diet effect: p<0.001, 41%), but was unaffected by strain. Placental expression of DMT1+IRE mRNA was significantly higher in RHL rats compared to Wistar rats (Figure 6B, strain effect: P<0.001), and was significantly increased in response to a low iron diet in both strains (Figure 6B, diet effect: P<0.001). Placental ferroportin protein expression was significantly higher in RHL rats compared to Wistar rats (Figure 6C, strain effect: P<0.05, 23%), but was unaffected by diet.

### Duodenal iron transport

Given the clear strain differences in maternal liver iron content, it was of interest to determine whether there was any difference in intestinal iron transport between the strains. When normalised to β-actin, expression of DMT1+IRE (RHL: 0.80 ± 0.12, Wistar: 0.76 ± 0.18), ferroportin (RHL: 1.17 ± 0.09, Wistar: 1.25 ± 0.11) and hephaestin (RHL: 0.81 ± 0.13, Wistar: 0.88 ± 0.11) mRNA in the duodenum did not differ between strains of rat when fed a control diet for 6 weeks in the non-pregnant state.

## Discussion

Long-term effects of iron deficiency during pregnancy on offspring blood pressure have been demonstrated previously in several strains of rat [10,12,17,18]. The long-term outcomes for the developing fetus are similar across these studies, and whilst it is assumed that these programmed attributes arise in response to identical maternal adaptations to the nutritional insult across all strains, this has never been directly assessed. This knowledge is required to improve understanding of the mechanisms of nutritional programming, which are currently thought to involve modification of fetal gene expression and resetting of organ structure, cellular function and endocrine regulation [21]. Previous work in the RHL strain identified alterations in iron homeostasis in response to a low iron diet during pregnancy [17,18]. This present study aimed to extend these findings to the more widely used Wistar strain of rat, and to examine any differences between the strains in terms of iron homeostasis and response to a low iron diet.

This study clearly demonstrates that the maternal and fetal livers are very sensitive to the feeding of a low iron diet prior to and during gestation. Additionally, whilst the total iron content of the placenta was unaffected by diet, a reduction in placental non-haem iron was observed in response to a low iron diet in both strains. These data are in good agreement with previous work with the RHL strain [7,17]. The reduction in maternal liver content in response to dietary restriction was associated with changes in the expression of key regulators of iron transport. Expression of the transferrin receptor (TfR) mRNA was up-regulated in the maternal liver by more than two-fold in both strains of rat. This is interpreted as a compensatory response to increase iron uptake into the liver cells and has previously been shown to be reversed by iron supplementation at selected points during pregnancy in the RHL [18]. Maternal hepatic hepcidin mRNA expression was vastly down-regulated in response to the feeding of a low iron diet in both strains of rat, almost to the point of being undetectable. Decreased hepcidin expression has previously been observed [17] and can be reversed by supplementation of iron during the second half of rat pregnancy [18]. As hepcidin prevents iron efflux [22,23], down-regulation may therefore contribute to meeting the greater requirements for iron during pregnancy.

Maternal ceruloplasmin mRNA expression was found to be unaffected by the feeding of a low iron diet, suggesting that transcriptional regulation is not necessarily related to the level of iron efflux from the cell. There has previously been contradictory evidence published regarding the sensitivity of ceruloplasmin to iron status [24-26]. However, in an *in vivo* rat model, there was no effect of iron status on plasma ceruloplasmin concentration or activity or hepatic mRNA expression [25]. Instead, it has been suggested that oxidase activity could be altered indirectly via changes in redox state [27]. Similarly to the maternal liver, TfR protein expression was increased in the fetal livers in response to prenatal dietary iron restriction, but only in the RHL strain. No change in expression of ferroportin was observed in either strain. However, the reduction in hepcidin mRNA expression in the fetal liver in response dietary iron restriction in both strains may modulate ferroportin internalisation via ubiquitination [28,29].

Placental expression of the transferrin receptor was increased in response to dietary iron restriction in both strains of rat, as previously observed in the RHL [17]. This may act to enhance iron uptake into the placenta and optimise transfer to the fetus. There was no associated increase in ferroportin protein expression, but its responsiveness to dietary iron status may instead be related to the observed reduction in fetal hepcidin mRNA expression modulating the internalisation of placental ferroportin [17,22,28]. In both strains of rat, placental DMT1+IRE mRNA expression was increased in response to dietary iron restriction, which would act to allow release of iron from intracellular vesicles. This is in agreement with previous work with the RHL [7], and suggests that adaptive mechanisms optimise the availability of transporters that allow iron to cross the placenta. However, it must be noted that work with a murine DMT1 knock-out model indicated that this transporter does not play an essential role in materno-fetal iron transfer and that another transporter must be responsible for iron uptake by fetal-derived cells [30].

Interestingly, there was considerable difference in liver iron content between the strains, with Wistar dams having approximately twice the quantity of iron present in their livers in comparison to RHL rats. This suggests greater promotion or efficiency of absorption and storage of iron in the maternal liver. Wistar dams also exhibited higher placental total and haem iron content in comparison to RHLs, which was associated with a greater placental weight. The opposite was observed in the fetal liver, where total iron content was significantly higher in fetuses of the RHL strain. This direct comparison of iron status between two different strains of rat highlights the care which needs to be taken when comparing findings from different experimental models and suggests that programmed phenomena, such as hypertension, do not necessarily arise from similar maternal adaptations to nutritional insults across strains or species. Whilst our research has identified common developmental processes and pathways which *are* affected across different strains and dietary insults during pregnancy, there may be other differences in response which impact on the progression of the programmed phenomenon [15]. Importantly, the liver iron content of the iron restricted Wistar rat was actually higher than the liver iron content of the *control* RHL rat, which demonstrates the inherent difficulty in labelling treatment groups as ‘deficient’ in comparison to an internal control in the absence of clinical thresholds.

The data suggests greater maternal to fetal iron transfer in the RHL rats, despite a reduction in placental weight in response to low iron diet in this strain only. This is consistent with the increased expression of placental iron transporters in this strain. Placental ferroportin protein and DMT1+IRE expression were significantly higher in rats of the RHL strain compared to the Wistar strain, which may account for a greater transfer of iron to the fetus at the expense of maternal liver iron stores. RHL rats also tended to have lower maternal liver TfR expression, although this marginally failed to reach statistical significance. Hepcidin mRNA expression was shown to be significantly lower in RHL fetal livers compared to Wistars, which may permit more ferroportin to be present at the membrane to facilitate increased transport of iron to other tissues. Unfortunately direct comparisons of the fetal liver iron transporters could not be made between strains, as expression of the normalising protein (tubulin) differed between strains, perhaps indicating structural (cytoskeletal) differences between liver cells of the two strains of rat. However, the lack of response of TfR protein expression to dietary iron restriction in Wistar fetal livers may explain the lower fetal liver iron content in this strain.

It has been previously shown that 72% of iron in the fetal liver is derived from maternal iron stores, and that the remaining 28% is from maternal dietary absorption [31]. We therefore went on to consider whether the differences in maternal iron status between the strains could be due to differences in absorption of iron from the diet in the duodenum. Iron absorption is known to be enhanced when the body is iron deficient [32], as indicated by increased expression of DMT1 in the duodenum [33]. However, no significant differences in duodenal DMT1+IRE, ferroportin or hephaestin expression were observed between the two strains of rat. The differences in maternal liver iron content cannot, therefore, be explained through differences in the expression of intestinal iron transporters, although a differential response to pregnancy cannot be excluded at this point. It is concluded that the differences in maternal liver iron content observed between the two strains are most likely a result of differences in the efficiency of placental transfer of iron to the fetus, as indicated by the greater fetal hepatic iron content and increased placental expression of iron export molecules observed in the RHL strain. Despite this, birth weight was reduced in response to iron restriction in the RHL strain only, indicating that the adaptive responses protected fetal iron status but not birth weight.

## Conclusions

Our findings demonstrate considerable differences in iron homeostasis between two strains of rat during pregnancy. While the impact of the iron deficient diet on maternal and fetal hepatic iron content was similar, the differences in baseline iron content are not. The results suggest that rats of the RHL strain promote greater transfer of iron across the placenta to the fetus in comparison to the Wistar rats, which maintain maternal iron status at the detriment of the fetus. The study suggests that whilst common developmental processes and pathways *are* affected across different strains and dietary insults during pregnancy [15], there may be other differences in baseline parameters or response to treatment which impact on the trajectory of the programmed phenomenon. This has important implications for studies of human health. Understanding how populations adapt to iron deficiency anaemia during pregnancy, and the impact of this on placental transfer and fetal iron status, is of considerable importance given the global burden of iron deficiency anaemia.

## Competing interests

The authors declare that they have no competing interests.

## Authors’ contributions

HM, LG, SM and SLE conceived and designed the experiments. RC and LG performed the experiments. RC, SM and SLE analyzed the data. RC, SM and SLE wrote the paper. All authors were involved in data interpretation. All authors read and approved the final manuscript.
